# Not just a vampire repellent: the adverse effects of garlic supplements in surgery

**DOI:** 10.1308/003588412X13373405385098

**Published:** 2012-09

**Authors:** A Lawn, P Sains

**Affiliations:** Western Sussex Hospitals NHS Trust

Apart from its culinary and mythical uses, garlic also has beneficial medicinal properties including antimicrobial, antiviral, antifungal and anti-cancer effects; and preventing heart disease by aiding the control of hypertension.^1^ Nevertheless, our experience has noted a deleterious effect from supplementary garlic consumption that has affected our clinical practice in colorectal surgery.

Garlic is known to be a blood thinner due its anti-platelet properties. Ajoene, a sulphur containing derivative of garlic, irreversibly inhibits platelet aggregation,^2^, potentiating anticoagulants such as aspirin, warfarin, dipyrimadole and clopidogrel.^3,4^ The composition of the garlic supplement affects its potency. Oil macerates have the highest content of ajoene and therefore exert the most potent effect.^5^

We performed elective high anterior resections for malignancy on two patients with no previous medical history, normal clotting profiles, platelet counts and not on anticoagulants or antiplatelets. Both laparoscopic cases converted to open due to the presence of generalised capillary ooze. Due to poor haemostasis, the risk of anastomotic leakage was high. Defunctioning ileostomies were formed despite original plans to perform a primary anastomosis. Retrospectively the patients admitted to self-medicating with garlic remedies. Recent recommendations from anaesthetic journals suggest that garlic supplements should be stopped seven days prior to operative intervention.^6^
